# Behavioral and physiological monitoring for awake neurovascular coupling experiments: a how-to guide

**DOI:** 10.1117/1.NPh.9.2.021905

**Published:** 2022-01-27

**Authors:** Qingguang Zhang, Kevin L. Turner, Kyle W. Gheres, Md Shakhawat Hossain, Patrick J. Drew

**Affiliations:** a The Pennsylvania State University, Center for Neural Engineering, Department of Engineering Science and Mechanics, University Park, Pennsylvania, United States; b The Pennsylvania State University, Department of Biomedical Engineering, University Park, Pennsylvania, United States; c The Pennsylvania State University, Graduate Program in Molecular Cellular and Integrative Biosciences, University Park, Pennsylvania, United States; d The Pennsylvania State University, Department of Neurosurgery, University Park, Pennsylvania, United States

**Keywords:** cerebrovascular physiology, functional brain imaging, sleep, movement

## Abstract

**Significance:**

Functional brain imaging in awake animal models is a popular and powerful technique that allows the investigation of neurovascular coupling (NVC) under physiological conditions. However, ubiquitous facial and body motions (fidgeting) are prime drivers of spontaneous fluctuations in neural and hemodynamic signals. During periods without movement, animals can rapidly transition into sleep, and the hemodynamic signals tied to arousal state changes can be several times larger than sensory-evoked responses. Given the outsized influence of facial and body motions and arousal signals in neural and hemodynamic signals, it is imperative to detect and monitor these events in experiments with un-anesthetized animals.

**Aim:**

To cover the importance of monitoring behavioral state in imaging experiments using un-anesthetized rodents, and describe how to incorporate detailed behavioral and physiological measurements in imaging experiments.

**Approach:**

We review the effects of movements and sleep-related signals (heart rate, respiration rate, electromyography, intracranial pressure, whisking, and other body movements) on brain hemodynamics and electrophysiological signals, with a focus on head-fixed experimental setup. We summarize the measurement methods currently used in animal models for detection of those behaviors and arousal changes. We then provide a guide on how to incorporate this measurements with functional brain imaging and electrophysiology measurements.

**Results:**

We provide a how-to guide on monitoring and interpreting a variety of physiological signals and their applications to NVC experiments in awake behaving mice.

**Conclusion:**

This guide facilitates the application of neuroimaging in awake animal models and provides neuroscientists with a standard approach for monitoring behavior and other associated physiological parameters in head-fixed animals.

## 1 Introduction

Although physiological studies using anesthetized animal preparations have advanced our understanding of brain function remarkably, there has been a push in the neuroscience community to utilize un-anesthetized animal models in neurophysiological experiments, given the disruption of normal physiology by anesthetics.^1^^–^^3^ Un-anesthetized approaches are particularly valuable to the neurovascular coupling (NVC) and functional neuroimaging communities, because the interpretation of both task-evoked and resting-state functional brain imaging data collected in un-anesthetized humans depend on our understanding of NVC under normal physiological conditions. In this paper, we will cover the importance of monitoring behavioral state in imaging experiments using rodents, drawing from examples in rodent literature, and then describe how to incorporate detailed behavioral and physiological measurements in imaging experiments.

Head-fixed preparations are invaluable for many physiological approaches,^4^^,^^5^ and offer the ability to limit behavioral complexity, which simplifies experimental design and data analysis, allowing researchers to focus on the behaviors of interest, such as locomotion,^6^^–^^8^ whisking,^9^ or licking.^10^ Head-fixed preparations also minimize motion artifacts and allow monitoring of neural activity and brain hemodynamics with two-photon laser scanning microscopy,^11^^–^^13^ functional ultrasound imaging,^14^ and high-density electrode arrays.^15^^–^^17^ In recent years, the availability of inexpensive, high-speed cameras, combined with powerful image processing algorithms, have enabled comparable advances in behavioral monitoring.^18^ Combining these techniques will provide more insights in understanding brain hemodynamics.

Recent studies have highlighted the importance of behavior and arousal levels in neural activity and the hemodynamics response. Fidgeting behaviors,^19^ including whisking and body motion, drive much of the spontaneous hemodynamics signal,^9^ and a re-analysis of “vasomotion” signals^20^ has shown a large component of spontaneous vascular signals are driven by body motions.^21^ These vascular dynamics are not due to changes in heart rate or other peripheral factors, as movement-driven arterial vasodilations are correlated with neural activity and blocked by local infusion of muscimol, indicating a local neural control.^7^ These movement-driven hemodynamic responses are not just an issue for studies in the somatosensory cortex. Large scale neural recordings have shown that whisking, and facial and body movements are robustly linked to brain wide changes in neural activity.^22^^,^^23^ In task-based studies, stimuli can introduce behavior changes locked to the stimulus. For example, visual stimulation in mice induces body motion that is highly correlated with the stimulus contrast,^24^ and auditory stimulation robustly induces whisking.^9^ Many other behaviors (blinking, sighing, and swallowing) can cause functional activation in multiple brain regions, and the frequency of these behaviors can be altered by arousal levels, age, and disease (see Ref. 19 for review). Behavior does not only drive vasodilation, it can also impact systemic blood oxygenation. Changes in respiration rate and phase are correlated with body movements,^25^^,^^26^ and increases in respiration rate drive increases arterial blood oxygenation that can increase tissue oxygenation independent of local vasodilation.^8^ As these changes in respiration are tied to arousal states,^27^^–^^29^ sensory stimulation,^28^^,^^30^^,^^31^ and motion,^32^ they can confound oxygen-sensitive measurements if not monitored.

In addition to fidgeting/movement-related brain dynamics, changes in arousal can cause large hemodynamic changes. In primates, decreases in arousal are associated with large increases in blood volume,^33^ and arousal transitions are associated with brain-wide hemodynamic signals.^34^^,^^35^ Sleep drives large, brain-wide increases in blood flow and volume,^36^^,^^37^ and the blood volume increase during rapid eye movement (REM) sleep can be several times larger than sensory-evoked response.^37^ In the absence of sensory stimulation, well-habituated mice frequently fall asleep,^37^ and head-fixed mice keep their eyes open during sleep,^37^^–^^39^ making detection of sleep more complicated than simply monitoring whether the eyes are open or closed. A careful monitoring of cortical state in experiments using “awake” animals is needed to elucidate potential confound in brain imaging studies.

Without monitoring behavior and arousal state, motion and arousal state signals will contribute to noise and variability in the physiological responses measured in experiments with un-anesthetized animals. The behavioral and arousal-liked changes can be much larger than any stimulus-evoked changes. Furthermore, as movement and sleep drive bilaterally symmetric signals that show substantially higher correlations than during rest,^36^^,^^37^^,^^40^^,^^41^ they will also contribute to “resting state” functional connectivity measures. Because of this, variations in the amount of a behavior will influence functional connectivity measures, and putative difference in connections across individuals or imaging sessions may simply due to differences in time asleep or moving.^33^^–^^35^^,^^42^

**Fig. 1:**
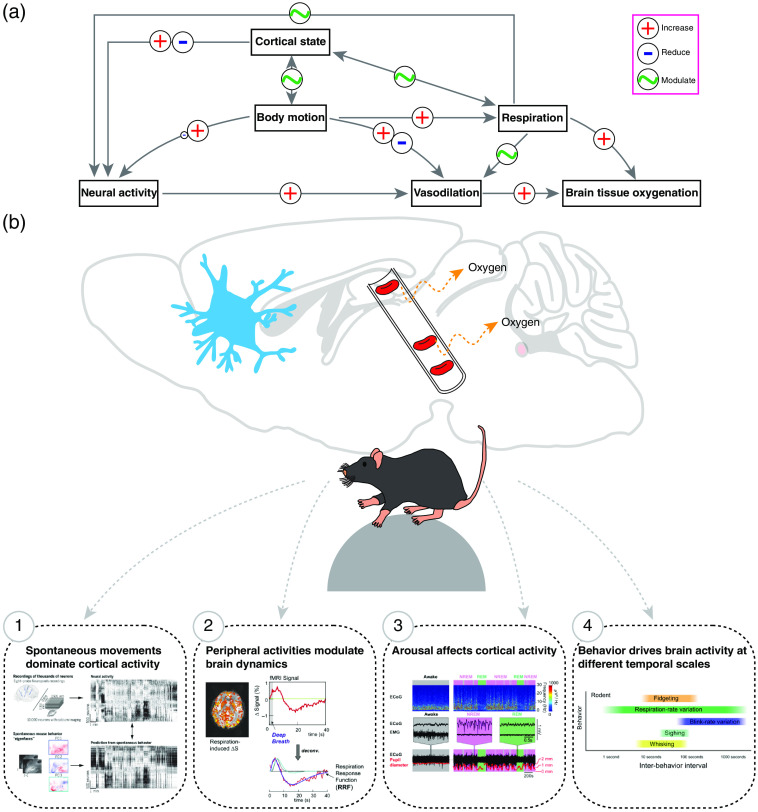
Overview of the relationship between movement, arousal, and neurovascular signals in rodents. (a) Schematic showing how the movement-related signals modulate the relationship between neural activity and brain hemodynamics. (b) Selected studies showing the obligation to monitor movement related signals. Data and figure are adapted from (1) Ref. 22, (2) Ref. 144, (3) Ref. 38, and (4) Ref. 19.

In this paper, we provide background for monitoring behavior and physiological signals from mice and other animals. Addition of behavior tracking and physiological measures of arousal state to neuroimaging experiments will help contextualize and classify brain imaging signals. This guide facilitates application of neuroimaging in awake animals and provides neuroscientists with a standard approach for monitoring the behavior and other physiological signals in head-fixed mice (Fig. 1). We believe that routine monitoring of these signals is essential for rigorous NVC research, to facilitate comparisons across laboratories, and to understand the physiological origins of neural and vascular signals.

## 2 Overview of the Procedures Described in this Tutorial

Here, we provide a how-to guide for monitoring and interpreting a variety of physiological signals [heart rate, respiration rate, electromyography (EMG), intracranial pressure (ICP), whisking, and other body movements] and their applications to NVC experiments in awake-behaving mice. For each physiological signal, we first provide a brief review of its effects (if any) on hemodynamics and electrophysiological signals, and summarize the measurement methods currently used in animal models, and then provide a step-by-step guide on how to incorporate these measurements with functional brain imaging and electrophysiology measurements. For data analysis, we supply example data sets and software for demonstration.

**Note**: the following tutorial is for a head-fixed mouse setup. Procedures involving live animals must be performed by trained experimenters and follow institutional guidelines, and local and national regulations. To ensure efficient neurophysiological measurements or successful behavior measurements under awake conditions, we recommend undertaking head-fixation habituation prior to the imaging session for a minimum of three days. We refer the readers to previously published descriptions of the surgical procedures for implanting headbars, windows, electrodes, and head-fixation in rodent models.^43^^–^^47^ Detailed plans for our head-fixation setup can be found here (https://github.com/DrewLab/Mouse-Head-Fixation), and the major parts and equipment used to build our optical imaging setups can be found here (Table S1 in the Supplemental Material). For measuring electrophysiological signals, we have found that battery-powered amplifiers (e.g., DAM80, World Precision Instruments) minimize power-line noise contamination of electrophysiological signals. In addition, as different signals come with drastically different temporal resolutions, when incorporate behavioral monitoring using videography, using cameras with transistor–transistor logic (TTL) ports able to trigger frame capture is essential. Moreover, due to the nature of data collection, some equipment may interact with the animal, such as the thermocouple used for respiration measurement. A steel protection tube, in combination with a compact manipulator, will provide precise positioning of the thermocouple and a stable signal over a long period.

## 3 Monitoring Large Bodily Motions in Awake, Head-Fixed Mice

**Fig. 2:**
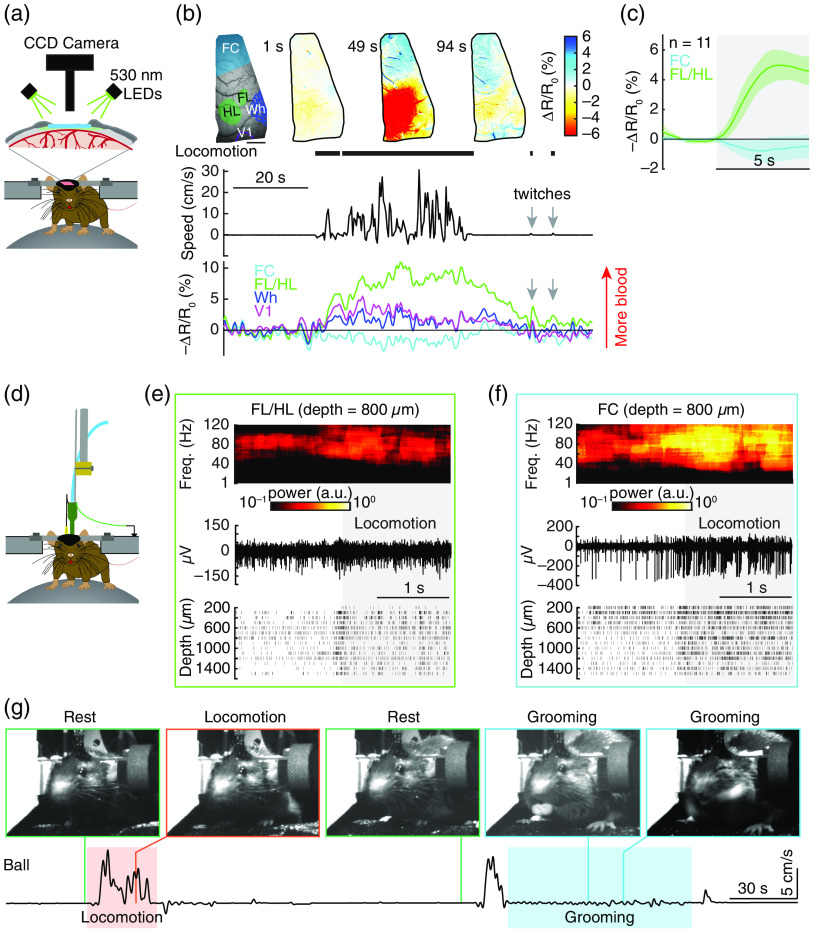
Locomotion drives global neural activity increase and cortical region specific hemodynamic responses. (a) Experimental setup for IOS imaging. Reflectance images are collected during periods of green LED light illumination at 530 nm (equally absorbed by oxygenated and deoxygenated hemoglobin, M530L3, Thorlabs). For these experiments, a CCD camera (Dalsa 1M60) is operated at 30 Hz with 4×4 binning (256×256  pixels), mounted with a VZM300i optical zoom lens (Edmund Optics). (b) Example showing cerebral blood volume (CBV) change during voluntary locomotion. Top left, an image of thin-skull window and corresponding anatomical reconstruction; scale bar = 1 mm . Top right, reflectance map before (1 s), during (49 s), and after (94 s) a voluntary locomotion event. Bottom, percentage change in reflectance ($-\Delta R/R_{0}$) during locomotion events for each brain region. Reflectance is inversely related to blood volume, and has been inverted for clarity. FC, frontal cortex; FL/HL, forelimb/hindlimb representation of the somatosensory cortex; Wh, vibrissae cortex; V1, visual cortex. (c) Population average of locomotion-triggered average of CBV (n=11 mice) responses in both FL/HL (green) and FC (blue). Data are shown as mean ± SD. (d) Experimental setup for neural activity measurements using multi-channel laminar electrodes. Neural activity signals are recorded using two linear microelectrode arrays (A1×16-3  mm-100-703-A16, NeuroNexus Technologies). The electrode array consists of a single shank with 16 individual electrodes with 100-μm interelectrode spacing. The signals are digitalized and streamed to SmartBox™ via a SmartLink headstage (NeuroNexus Technologies). The arrays are positioned perpendicular to the cortical surface, one is in FL/HL and the other one is in FC on the contralateral side. (e) Example trial showing the large increase in gamma-band LFP power (top), raw signal (middle), and spike raster (bottom) during locomotion from a site 800  μm below the pia in FL/HL. Shaded area indicates the time of locomotion. (f) As in (e) but for FC. (g) Identifying grooming events from analog signals from rotary encoder. Subpanels (a)–(f) are adapted from Ref. 8.

Awake animals move, both spontaneously^8^^,^^9^^,^^19^^,^^41^ and in response to sensory stimuli.^9^^,^^24^ These movements (during which the animal is able to move its limbs and that we colloquially refer to as locomotion), generate complex changes in neural activity in many brain regions,^48^ not just somatosensory and motor regions^8^^,^^41^^,^^49^^–^^55^ [Figs. 2(d)–2(f)]. In some brain areas, the increases in neural activity is accompanied by increase in blood flow,^8^^,^^40^^,^^41^^,^^51^^,^^56^ but decrease in blood flow in other cortical regions.^8^^,^^41^ In somatosensory areas, the changes induced by locomotion and movement are comparable to those generated by sensory stimulation.^9^ Locomotion drives increases in blood flow and volume in the cerebellum,^56^ somatosensory^8^^,^^41^^,^^51^ and visual cortex,^8^^,^^41^ and hippocampus.^40^^,^^57^ These increase in flow are not driven by increase in heart rate or blood pressure,^8^^,^^51^ as they are blocked when local spiking activity is blocked.^7^^–^^9^ Even short movements (twitches or brief postural adjustments) can drive robust hemodynamic signals [Fig. 2(b)]. If these ubiquitous movements are not monitored, the changes in hemodynamic signals accompanying them may be erroneously interpreted as “noise” or vasomotion.

For imaging experiments, mice can be either head-fixed on a spherical treadmill^7^^,^^8^^,^^19^^,^^41^^,^^50^^–^^52^^,^^58^^–^^76^ [ Fig. S1(b) in the Supplemental Material] or a rotating disk^77^^–^^80^ which allows them to freely locomote. Alternatively, mice can be head-fixed with their body in a tube [Fig. S2(b) in the Supple-mental Material], which is compatible with fMRI measures,^3^ whisker-based tactile behaviors^9^^,^^45^ and the study of sleep.^37^^,^^81^ The techniques for monitoring large bodily motions are different in each paradigm, and we describe them in turn.

### 3.1 Detection of Movement on a Spherical Treadmill

The standard spherical treadmill in our lab is made from a plastic ball 6 cm in radius (Kaytee clear run-about exercise ball, 5 in.). To give the mouse better traction, we wrap the path of the mouse on the ball with grip tape (3M 310 Safety-walk, S-16032, McMaster-Carr). The mouse and the headbar holder are positioned so that the back of the mouse’s head is ∼1.5  cm forward and 2 cm above the highest point of the ball. This positioning allows the mouse to easily stand, walk, or groom, but may need to be adjusted depending on the size of the mouse. A metal shaft (1/4 in. in diameter and cut to 7 in. in length, 1327K113, McMaster-Carr) is inserted through the ball and attached to a rotational encoder (E7PD-720-118, US Digital) to monitor rotational velocity. The position of the ball is secured with a shaft collar (9414T6, McMaster-Carr) on each side. The encoder and the other end of the shaft are attached to an optical breadboard (12×12×1/2  in., MB12, or 8×8×1/2  in., MB8, Thorlabs). Mounting the entire apparatus on a breadboard facilitates movement and adjustment of the head-fixation setup, and this apparatus can be easily inserted under two-photon microscope or other optical imaging systems (Fig. S2 in the Supplemental Material).

The rotatory encoder outputs an analog signal, which is proportional to the angular velocity of rotation [Fig. 2(b)]. Using analog motion detectors has some advantages over video monitoring as the data takes up much less space and the analysis is much simpler. We acquire the analog signal and filter it offline with 10-Hz zero-lag low-pass filtering (fifth-order Butterworth filter) in software. To detect movements of the mouse, we use an acceleration threshold. Acceleration is calculated as the first derivative of the filtered velocity signal. The absolute value of the acceleration is then categorized as locomotion if the absolute value of the acceleration is above a threshold of $0.03\;m/s^{2}$, and resting if the absolute value of acceleration is less than this threshold. To align the locomotion signal with other measurements, such as functional brain imaging signal, which has different temporal resolutions, we characterize that the image frame is collected during locomotion if at least 10% of the instantaneous acceleration is above the threshold within the time spanned by continuous imaging frames. Also, if two consecutive locomotion events occur within 1 s of each other, the time elapsed in between is considered as a continuous locomotion period.^6^^–^^8^^,^^41^^,^^51^^,^^59^^–^^61^ We have found that this heuristic allows us to detect both long walking bouts and short “twitches” accurately [Fig. 2(b)], as both kinds of movements are associated with robust vasodilation in the somatosensory cortex [Fig. 2(b)]. In our experience,^8^^,^^41^^,^^51^ the absolute velocity of the locomotion does not impact neural or hemodynamic activity, only the state of motion itself. Neural and hemodynamic responses evoked by motion can be aligned to motion onset to generate a “locomotion-evoked average” or “locomotion-triggered average,” just as is done with stimulus-evoked responses. These locomotion-evoked averages, at least in the somatosensory cortex, are highly repeatable across locomotion events of comparable duration [Fig. 2(c)], and are stable over months of imaging.^59^ Notably, brief body motions (twitches) can drive robust changes in blood volume [Fig. 2(b)]. While the amplitude of the hemodynamic responses increases with increasing event duration, this is largely due to vasodilation of veins which dilate slowly over tens of seconds,^82^ as the arterial dilation reaches its maximum within a few seconds.^59^ Lastly, grooming events can be distinguished from locomotion by the oscillatory signal in ball velocity with no net directed motion [Fig. 2(g)].

### 3.2 Detection of Large Bodily Movements in Restrained Mice

For experiments where locomotion is not wanted, mice can be placed in a plastic cylinder for imaging [Fig. S2(b) in the Supplemental Material]. The mouse is inserted into an acrylic tube (5 in. long, 1.5 in. outer diameter, 1.375 in. inner diameter, McMaster-Carr,8585K207), with the mouse head extending out, allowing the mouse to use its front paws to grip the tube edge [Fig. S2(b) in the Supplemental Material]. The tube is attached to the customized holder via Velcro tape. Typically, the head bar is about 3 cm above the bottom of the body tube. After head-fixation, the mouse should be crouching in a natural position in the body tube, with its paws resting on the edge of the tube. To measure body movement while the mouse is head-fixed in a plastic tube, a force sensor (Flexiforce A201 sensor, Tekscan, Boston, Massachusetts) is placed below the plastic tube to detect body movement.^9^^,^^37^ The signal is amplified 1000× (Model 440, Brownlee Precision), zero-lag low-pass filtered (<20  Hz, second-order Butterworth filter), and digitized at 20 kHz. Similar to the detection of locomotion, changes in force that exceeded a threshold were flagged as body movements by the animal.

## 4 Video Monitoring of Behavior

### 4.1 Monitoring Whisker Movement

**Fig. 3:**
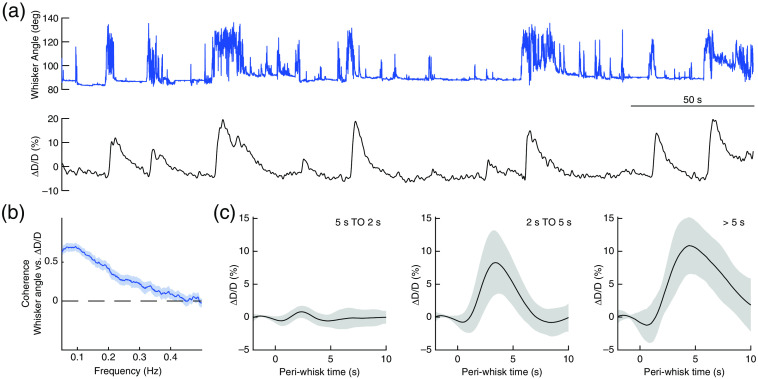
Volitional whisking drives arterial dilation. (a) Top, whisker angle. Bottom, pial arterial diameter changes (ΔD/D) associated with volitional whisking. Note even short whisking events cause significant arterial dilation. (b) Coherence between whisking and pial arterial diameter oscillations. (c) Arterial diameter responses to brief (0.5 to 2 s, left), moderate (2 to 5 s, middle) and extended (>5  s, right) volitional whisking events. Adapted from Ref. 21.

The rodent vibrissae system is an extensively studied experimental model of sensorimotor processing.^83^^,^^84^ Whisker sensory processing occurs in a highly distributed manner in the mouse brain. Even a brief deflection of a single whisker can evoke signals in many brain regions downstream of somatosensory cortex.^85^ The rodent whisker-to-barrel cortex also is a well-established sensory system to investigate NVC. Movement of whiskers in awake^9^^,^^86^ and anesthetized rodents^82^^,^^87^^–^^93^ cause corresponding changes in neural activity and hemodynamic signals (Fig. 3), and these signals will not be restricted to the somatosensory cortex, but will be present throughout the brain.^22^^,^^94^

Not only is whisker movement of interest to the researcher who wishes to understand whisker sensory processing (and sensory system in general), but these movements provide data about the internal state of the animal.^95^ Although the whiskers are held still while the mouse is at rest, the mice do volitionally whisk. Volitional whisking will bilaterally increase firing rates in brainstem, thalamic, and cortical motor somatosensory region.^96^^–^^99^ Whisking can also be evoked by sensory stimulation, even if the stimuli are not directed at the whiskers, such as auditory stimulation.^9^

**Fig. 4:**
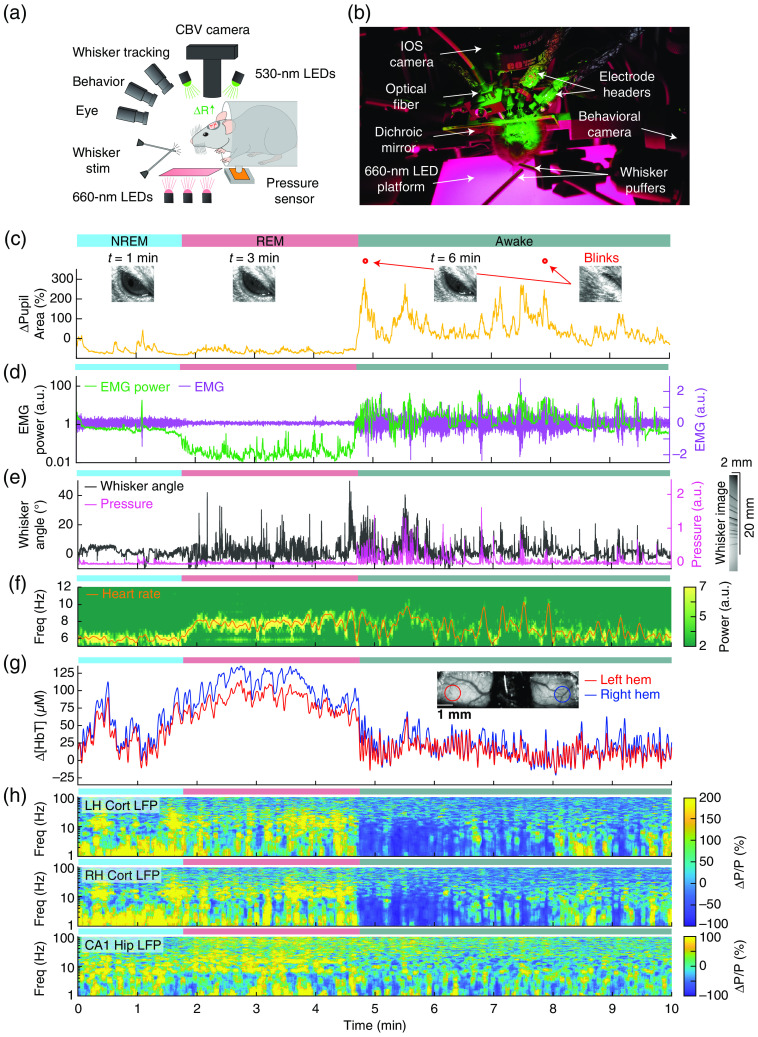
Cortical state alterations drive large hemodynamic signals in the brain. (a) and (b) IOS imaging and behavioral monitoring of a restrained mouse in a plastic tube. The brain is illuminated with 530-nm LEDs, and changes in reflected light are captured by a CCD camera mounted above the head. Other cameras track the whiskers (illuminated by 660-nm LEDs beneath the animal), the eye (illuminated by 780-nm LEDs), and changes in animal behavior. A piezo sensor to record changes in body motion is located beneath the animal, which rests head-fixed in a cylindrical tube. Tubes direct air to the distal part of the whiskers (but not the face), and do not interfere with volitional whisking. Changes of (c) pupil size, (d) EMG, (e) whisker angle and motion, (f) heart rate, (g) total hemoglobin, and (h) spectrogram of cortical (LH Cort LFP and RH Cort LFP) and hippocampal local field potential (CA1 Hip LFP) during cortical state transitions. It is noteworthy that the blood volume during sleep is greater than during awake, and the fluctuations during NREM are much larger than when the mouse is awake. Adapted from Ref. 37.

While whisker movement can be detected with video monitoring of the face,^22^ more precise quantification of whisker movement requires short exposure time, higher frame rate cameras. In our lab, whisker movements [Figs. 3(a) and 4(e), and Fig. S2(d) in the Supplemental Material] of un-anesthetized mice are captured at 150  frames/s with a Basler A602f camera with an 18-mm DG series FFL lens (#54-857, Edmund Optics). The whiskers are diffusely illuminated from below with 625-nm light (Edmund Optics, #66-833) or 780-nm light (for two-photon laser scanning microscopy, 2PLSM; M780L3, Thorlabs). Either a ground glass diffuser or several sheets of paper can be used to make the illumination of the whiskers more homogenous [Fig. 4(b) and Fig. S2(b) in the Supplemental Material]. We capture a small region of interest (ROI), typically 30×350  pixels, corresponding to a ∼2-mm×∼24-mm field of view, which is adequate to detect whisker angle [Fig. 4(e)]. The ROI needs to not contain the face, and should be homogenously illuminated. The average angle of all the whiskers is then quantified using an algorithm that makes use of the Radon transform to detect the overall angle of the whiskers (https://github.com/DrewLab/Whisker-Tracking), as can be done with line scans of single capillaries to determine red blood cell velocity.^100^ The peaks of the sinogram correspond to the position and the angle of the whiskers in the image. The average whisker angle is extracted as the angle of the sinogram with the largest variance in the position dimension. For tracking the entire length of the whisker and its dynamic interactions with objects, more sophisticated software is needed.^101^^–^^103^ Vibrissae angles from any dropped camera frames are filled by linear interpolation between the nearest valid points. Whisker angle is low-pass filtered (<20  Hz) using a second-order Butterworth filter. To align with functional brain imaging measurements, whisker angle data are then resampled down to the imaging frame rate (for our experiments, 30 Hz). To identify periods of whisking, whisker acceleration is obtained from the second derivative of the position and binarized with an empirically chosen acceleration threshold for a whisking event. Acceleration events that occur within 0.1 s of each other are considered as a single whisking bout. As with locomotion-triggered average, the hemodynamic responses induced by whisking can be visualized by aligning the hemodynamic signals to whisk onset to generate a whisking-triggered average^9^^,^^37^ [Fig. 3(c)].

### 4.2 Video Monitoring Body Motions and Postural Adjustments

Optical imaging studies and fMRI studies in awake animals rely on head and/or body restraint^1^ to minimize head motion. With this setup, rodents are often imaged on top of a treadmill or in a tube, which allows a great deal of body motion. If only ball motion or pressure sensor signals are monitored, other movements (i.e., stomping, grooming, and twitching)^19^^,^^104^ may not be detected unless they drive appreciable change of ball rotation (as shown in Fig. 2) or pressure sensor signals (as shown in Fig. 4). These relatively small motions are accompanied by robust motor cortex activation and drives neural activity signal and brain hemodynamics signal change.^9^^,^^19^^,^^105^^–^^109^

**Fig. 5:**
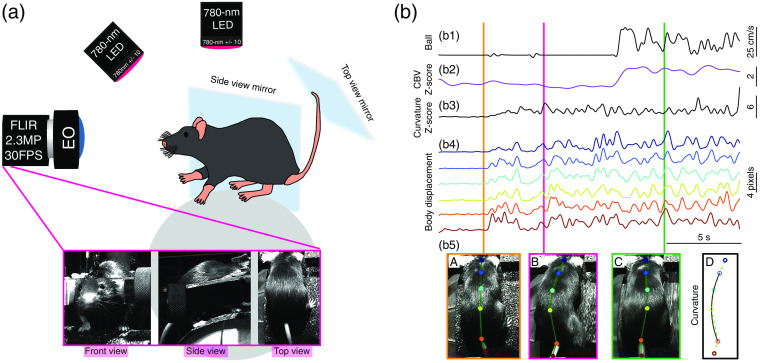
Monitoring of small body movements using high-speed camera and computer vision. (a) Experimental setup. Different body parts can be monitored from different angle (front, side, and top views) with one single camera using reflected images from mirrors. To monitor the trunk movements, a top view mirror can be placed above the mouse and tilted at a 45-deg angle. To monitor the forepaw, hindpaw and tail movements, a side view mirror can be placed on the side of the mouse and slightly rotated to ensure the image of these body parts captured by the camera. (b) Track body positions with DeepLabCut. (b1) Ball velocity from the optical encoder. (b2) CBV changes in response to locomotion recorded with fiberphotometry. (b3) Spine curvature changes during rest and locomotion. It is noteworthy that the spine curvature changes when the mouse makes any movement regardless of the ball velocity. (b4) Displacement of each marker between consecutive frames. The displacement is calculated as the Euclidian distance of a dot in two subsequent frames, which reflects the body movement. (b5) Position of the spine at different times during the experimental session. Subpanel A shows the spine position during rest. Subpanel B shows the spine position when the mouse adjusts the posture with minimal treadmill movement. Subpanel C shows the spine position when the mouse is running. Subpanel D shows the calculation of the spine curvature.

In addition to fidgeting- or stimulus-evoked movements, head-fixed mice rest their body at different postures, especially on the setups which allow more body motion. At rest, some mice bring feet close together and arch their back, or twist their body to maintain certain posture, resulting in a change of curvature of spine column (Fig. 5). As in pathological conditions, the cervical curvature change significantly affects the vertebral artery blood flow, the normal change of spine column may also contribute to the brain hemodynamics regulation. Animal body posture may also affect brain hemodynamics through multiple mechanisms, e.g., the ICP changes^110^^,^^111^. Also, different static body posture will set a distribution of tonic muscle activity and proprioceptive input.

Advances in computer vision and deep learning^18^^,^^112^ have made the detection and quantification of these body motions easier. Combining high-speed cameras with those advanced algorithms allows very sensitive body and limb movements tracking.^9^^,^^76^^,^^113^ Here, we provided a simple example of body position and spine/tail curvature tracking using DeepLabCut.^18^^,^^112^ For behavior imaging, we use a camera (FLIR blackfly USB camera) running at 30  frames/s. The camera can be placed in the front or over the head of the setup, based on the experimental setup. To capture the entire body of the mouse (front view, top view, side view, and back view), instead of using multiple cameras (which dramatically increases the amount of data and complexity), we use mirrors placed at different angles to reflect different body parts and use one single camera with a wide-angle lens to capture the reflected images [Fig. 5(a)]. The infrared light is diffused to ensure an evenly distributed illumination. A shorter exposure duration combined with brighter illumination ensures videos without blur. Before analyzing the videos in DeepLabCut,^18^^,^^112^ we crop the videos to include only the ROI, making it easier to produce better tracking performance with fewer training images. By combining movement velocity, movement direction with brain hemodynamics and electrophysiological signals [Fig. 5(b)], we can better understand movement-specific brain functions, and quantitatively understand the fidgeting contribution to brain signals.

Note: Since image segmentation and image analysis rely on high-contrast image sequence, the choice of colors for background and the mouse strain can affect image quality. Behavioral illumination should occur with infrared light so as not interfere with any acquired fluorescence/intrinsic optical signals (IOSs), and because rodents are blind to it. The animal may move perpendicular to the focal plane, e.g., the hips and tail are different distances from the camera. To maintain image focus, a lens with an adjustable f-stop should be used to maximize the focal depth by reducing the aperture size. However, this increased focal depth comes at the expense of light gathered. If the camera exposure time is not short enough, frames in the image sequences will have blurry objects, e.g., the whiskers, as they move very rapidly. This combination of increased depth of field and short exposure time requires sufficient ambient lighting to generate quality images. No matter how good the computer vision algorithm is, it will be difficult for it to identify certain body parts from blurry images. We also recommend the use of high-resolution video for better spatial resolutions (high-definition is recommended for fast-moving body parts). But for whole-body assessment while the mouse is resting, low and medium-quality videos are adequate.

### 4.3 Monitoring Pupil Diameter

Pupil diameter has been widely applied to monitor cortical state during locomotion,^65^^,^^114^^–^^116^ or other small movement,^117^ tactile detection task,^118^ and sleep transition,^38^ as changes in pupil diameter are thought to correlate with the activity of neuromodulators, including noradrenaline (NA) and acetylcholine (ACh) [see Ref. 119 for review]. We monitor the pupil using video monitoring. A Basler A602f camera with a fixed focal length lens (#67-714, Edmund Optics) or a telecentric lens (#58-430, Edmund Optics) is used to image the pupil at 30  frames/s (200×200  pixels). The camera is positioned 30 cm away from the mouse. The eye is illuminated with 780-nm light (M780L3, Thorlabs). Illumination was done at an angle of 60 deg relative to the axial midline of the mouse so that the reflection of the light off the cornea does not interfere with pupil visualization. We segment the images of a black pupil on a gray iris background with a sequence of image processing manipulations done with a custom-made MATLAB script (https://github.com/DrewLab/Pupil-Tracking). For other protocols for performing pupilometry, see Ref. 120.

**Fig. 6:**
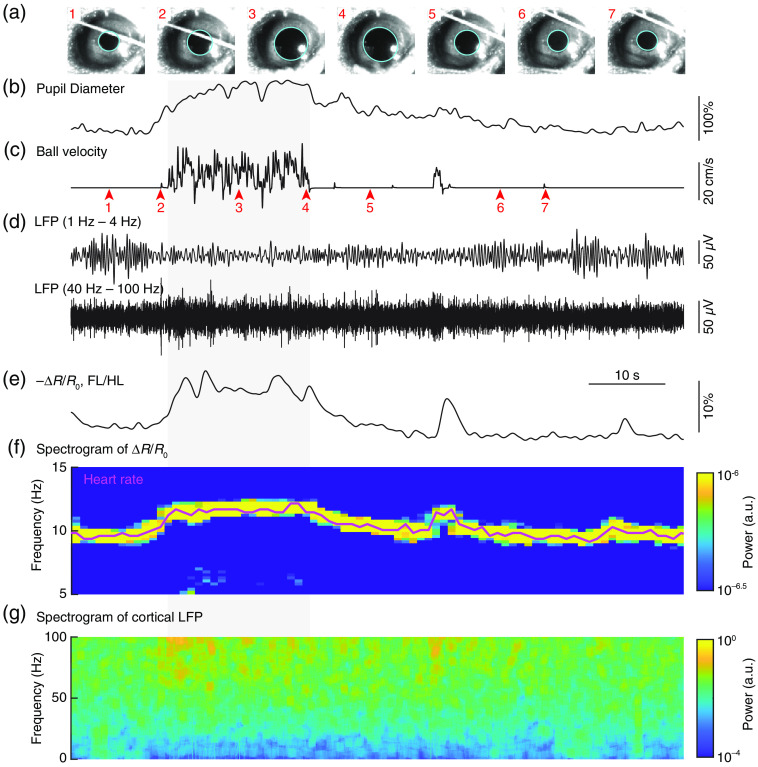
Monitoring cortical state change during locomotion. (a) Video frame images of the mouse’s eye (1 to 7) are shown where acquired at the times indicated in the pupil recording trace. Pupil diameter is recorded on video and extracted offline via a fitted ellipse (cyan). (b) Pupil diameter in percentage change is shown as a function of time. (c) Locomotion speed is indicated by rotary encoder. Long bout of locomotion period is indicated by gray shading. (d) Alpha- (1 to 4 Hz) and gamma-band (40 to 100 Hz) LFP recorded from somatosensory cortex. (e) Percentage change in reflectance ($-\Delta R/R_{0}$) during locomotion events in the forelimb/hindlimb representation of the somatosensory cortex (FL/HL). (f) Extract heart rate information from optical intensity fluctuations. (g) Spectrogram of cortical LFP signal. It is noteworthy that the increase in gamma-band (40 to 100 Hz) power and decrease in low frequency (<10  Hz) power during movement.

During wakefulness, pupil dilation is correlated with whisker and body movements, and increase in cerebral blood volume (CBV) (Figs. 4 and 6). Pupil diameter is qualitatively different during sleep. During REM sleep, it remains mostly constricted, while during non-rapid eye movement (NREM) sleep, the pupil’s diameter fluctuates, though the diameter is smaller than awake states (Fig. 4).^38^

## 5 Electromyography for Monitoring Muscle Tone

**Fig. 7:**
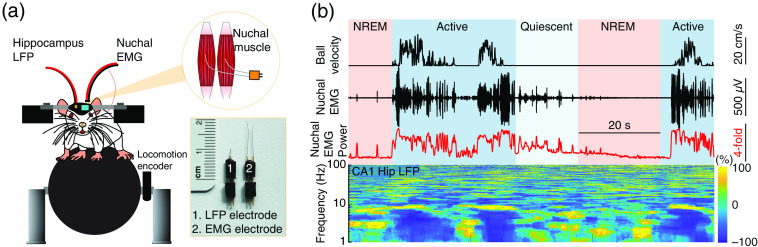
Monitoring hippocampus LFP and nuchal muscle EMG during resting, sleeping and during locomotion. (a) Experimental setup. A tungsten stereotrode is implanted ∼1500  μm below the pia into the CA1 region of the hippocampus. Stainless-steel EMG wires are implanted into the nuchal muscle, and the entire area is sealed with dental cement. The inset shows the surgical procedure and the electrode fabrication. (b) Nuchal EMG and hippocampus LFP dynamics during cortical state transitions. The bottom panel shows the spectrogram of hippocampus LFP during cortical state transitions.

Not all body motions are detectable with video. For example, head-fixed animals do not move their head, but they can exert force on the headbar.^121^ Preceding, accompanying, or following gross motor movements or small posture changes, multiple muscle groups become engaged, e.g., the neck muscle.^122^^,^^123^ Electrical monitoring of muscle activity (EMG) allows measurements of the activity of single muscles with high temporal resolution. Immediately before and during locomotion and other movements, EMG increases in head-fixed adult mice^73^ and neonate rodents.^124^ EMG increases in muscle groups precede locomotion initiation. In humans, visual stimuli produce stimulus-locked responses in limb skeletal muscle EMG.^125^^,^^126^ Aside from stimulus-evoked responses, during rest, muscle tone also changes in association with arousal state [Figs. 4(d), 7(b), and Fig. S2(c) in the Supplemental Material]. EMG also allows measurement of muscle tone, which is a key component of sleep scoring in animal models, and can be used to differentiate stages of sleep (such as NREM and REM) from periods of awake quiescence. For example, during active sleep, the nuchal muscle and forelimb muscle become atonic, and EMG power recorded from nuchal muscles decreases in both adult^37^ and neonatal rodents.^81^^,^^124^^,^^127^

For detailed theoretical, technical, and practical aspects of EMG recordings, we direct the reader to the excellent tome by Loeb and Gans.^128^ While the advances in microelectronics that have driven large scale electrophysiology in the brain have been applied to EMG technology,^129^ here we focus on using more conventional EMG techniques to detect muscle activity and arousal state, and provide examples of measuring EMG from nuchal muscle for monitoring muscle tone during sleep-wake transitions. We make recordings of nuchal muscles as they are accessible and close to the headbar, facilitating an easy mounting of the connector.

### 5.1 Electrode Fabrication

For EMG recording in nuchal muscle, stereotrodes [Fig. 7(a)] are constructed with PFA-coated 7-strand stainless-steel microwires (#793200, A-M systems, Sequim, Washington). EMG stainless-steel microwires are threaded through polyimide tubing (#822200, A-M Systems, Sequim, Washington) giving an interelectrode spacing of several millimeters. The stainless steel microwires are crimped to gold pin connectors (#520200, A-M Systems), with impedances typically between 70 and 120 kΩ at 1 kHz. About 3 mm of the coating at the tip of the EMG electrodes is stripped off with sharp forceps before implantation.

### 5.2 Monitoring Neck Muscle EMG

The skin above the neck is resected and the electrodes are implanted by inserting the EMG wires into each nuchal muscle for EMG recording. The skin is then re-attached to the edge of the occipital bone (VetBond, 3M, St. Paul, Minnesota), and the entire area is sealed with dental cement [Fig. 7(a)]. Electrical activity from the nuchal (neck) muscles is amplified and digitally bandpass filtered (300 to 3000 Hz) using a third-order Butterworth filter. To obtain the power of the EMG [Figs. 4(d), 7(b), and Fig. S2(c) in the Supplemental Material], the signal is squared and then convolved with a Gaussian kernel (0.5 s standard deviation).

### 5.3 Interpreting EMG Activity

EMG power will vary over several orders of magnitude during transition from awake to different sleep states. For visualization, it is helpful to plot the EMG power (300 to 3000 Hz) on a log scale. In awake animals, there is always tonic muscle tone, which decrease approximately 20-fold during NREM sleep in mice, and almost completely suppressed during REM sleep [Figs. 4(d), 7(b), and Fig. S2(c) in the Supplemental Material].

## 6 Other Physiological Signal Monitoring

An efficient, fine-tuned interplay between the brain and body (central and peripheral nervous systems) via neuronal, vascular, and humoral mechanisms is essential to maintain bodily functions and homeostasis. A disturbance of brain-body interactions is a major contributor to many diseases affecting the brain, heart, liver, and metabolism. The changes in posture, small behavior, or brain states (e.g., sleep) will cause changes in physiological signals, such as heart rate, respiration, muscle tone, and ICP, which will directly/indirectly cause changes/fluctuations in electrophysiological or hemodynamic signals.

### 6.1 Monitoring Heart Rate Changes during Behavior

The heart rate is not static, and it is increased by exercise [Fig. 6(f)], stress, and sensory stimulation, and decreases during immobility and sleep [Fig. 4(f)]. In mice, telemetric recordings in the home cage over the course of the day have shown that heart rate averages ∼12  beats/s.^130^ This includes periods of rest and movement, so heart rates above and below 12 Hz are well within the normal range. There are also strain and age-dependent differences across mice that can be substantial.^131^ The potential impact of systemic variables needs careful consideration in brain imaging studies. For some imaging modalities, such as BOLD fMRI, heart-rate fluctuations can be a significant source of noise,^132^ as the fluctuations are near the sampling frequency, leading to aliasing. For others, such as IOS imaging, the heart rate-induced fluctuations in the signals are orders of magnitude smaller than sensory-evoked ones.^9^ Heart rate, and the associated changes in blood pressure affect various vessel types differently. As arteries have active autoregulatory responses, while veins passively change their diameters in response to pressure changes, arteries and veins will be affected by blood pressure differently. Pharmacologically disrupting normal cardiovascular changes accompanying voluntary locomotion significantly affects the hemodynamic responses in veins, but not arterioles.^51^ This suggests that normal venous distention accompanying locomotion requires normal heart rate modulations. To an even finer scale, the pulsation of the heart cause pulsations in brain tissue, leading to image distortion and loss of resolution,^133^ which may limit our ability to study both physiological responses and pathological changes in the intact central nervous system. Heart rate can be invasively monitored using electrodes.^133^^,^^134^ However, in many cases, heart rate can be extracted from IOS images^8^^,^^9^^,^^51^ or line scans from capillaries.^43^^,^^135^

**Fig. 8:**
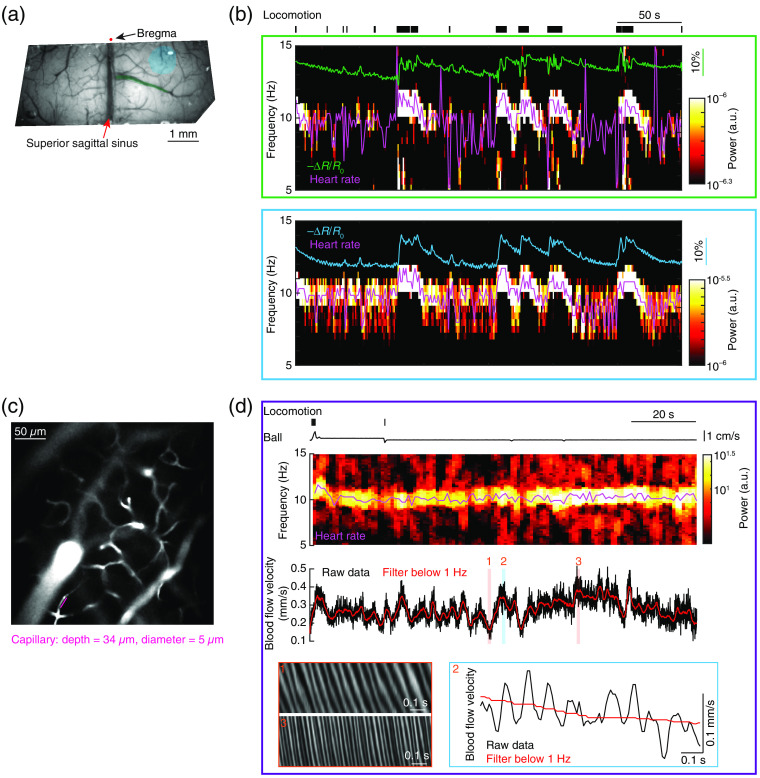
Extracting heart rate information from functional brain imaging signals. (a) Image showing a polished and reinforced thin-skull window covering the somatosensory cortex of both hemispheres. (b) Spectrogram of optical intensity changes during locomotion using the pixels covering mainly draining vein (top, green box) and mainly pial arterioles and brain parenchyma (bottom, cyan box). The magenta traces indicate the extracted heart rate information. The green and cyan traces indicate the inverse of optical intensity changes ($-\Delta R/R_{0}$) during locomotion. (c) The vasculature around the measurement sites, as indicated by the magenta line. (d) Instantaneous blood flow velocity and its spectrogram during locomotion. Shaded areas 1, 2, and 3 in the instantaneous blood flow velocity trace indicate space-time plots of 1 second line scan data for the capillary (1 and 3), and the calculated instantaneous velocity (2). Panel (c) is adapted from Ref. 6.

Heart rate information can be extracted from intrinsic optical signals. We direct the reader to the following references for details on how to implant cranial^46^ or thin-skull windows.^43^^,^^44^ To avoid aliasing of the heart rate signal, images must be acquired at greater than twice the highest heart rate. We use a 30  frames/s acquisition rate with isobestic (530 or 570 nm) illumination. An ROI avoiding large draining veins is drawn for further time-frequency analysis [Fig. 8(b)]. While the amplitude of the heart rate-related pulsations is small (they typically have a peak to peak $\Delta R/R_{0}$ of 0.02% to 0.1%), and much smaller than sensory- or locomotion-evoked changes [Fig. 2(c)] that can be 1% to 5% or even more, they have a high signal to noise. To extract heart rate-related information, we first take the temporal derivative of the median window reflectance to remove the prominent low-frequency oscillations in the IOS. We then calculate the spectrogram using Chronux Toolbox^136^^,^^137^ with a sliding window of 2 s on the median window reflectance. In the 5 to 15 Hz frequency band, the heart rate driven oscillations show up as a prominent peak [Fig. 8(b)], which can be detected by finding the frequency with the maximum power in a given time window. Under normal physiological conditions, the heart rate will vary with time. This is known as heart rate variability, and is indicative of normal fluctuations in sympathetic and parasympathetic drive. Heart rate increases (up to ∼12  Hz) when the animals locomote [Figs. 6(f) and 8(b)], and is substantially lower in NREM sleep than in the awake state [Fig. 4(f)].

Heart rate information can also be extracted from capillary line scans. The velocity of red blood cells in capillaries is significantly affected by the heart beat oscillations, and show oscillations at the heart rate frequency. Power spectral analysis of the red blood cell velocity of brain capillaries show a significant peak around heart beat frequencies^43^^,^^135^ [also see Fig. 8(d), inset 2]. Therefore, applying spectrogram analysis using Chronux Toolbox^136^^,^^137^ used for IOS, we can also extract heart rate information from capillary blood flow velocity signals [Fig. 8(d)]. As capillary blood flow is affected by local regulation^138^^,^^139^ and intravascular factors,^140^ when extracting heart rate information, one should take caution to make sure the blood flow is free of any obvious stall events,^6^^,^^141^^,^^142^ which is mainly driven by leukocyte adhesion.^143^

### 6.2 Monitoring Respiration during Behavior

Like heart rate, the respiration rate varies dynamically depending on the arousal state of the animals. Respiration rate plays an important role in determining oxygenation of brain tissue, as respiration rate can explain about as much variance in oxygenation as neural activity does.^6^^,^^8^ Cognitive tasks have been shown to drive stimulus-locked respiration in humans.^31^ Respiration-entrained local field potential (LFP) oscillations are observed brain-wide and in many species.^29^ Early fMRI studies revealed that the global signal recorded through fMRI is affected by breathing,^144^^–^^146^ and the signal is also affected by variations in respiration during resting-state studies.^32^^,^^147^^,^^148^ Recent studies have also shown that brain tissue oxygenation is greatly affected by respiration during voluntary locomotion in mice.^8^^,^^149^ Moreover, as an important waste clearance pathway, cerebrospinal fluid (CSF) flow in the brain and spinal cord is dramatically affected by respiration.^150^^–^^152^ Breathing rapidly is often associated with fear or alertness, and it may also serve an important role in stimulating brain areas responsible for information processing, which facilitates faster responses to environmental stimuli. There are reciprocal connections between the respiratory nuclei and the locus coeruleus^27^ and other brain regions involved in arousal.^25^ Because of this, respiration can be used as an important signal for cortical state segregation.^153^

**Fig. 9:**
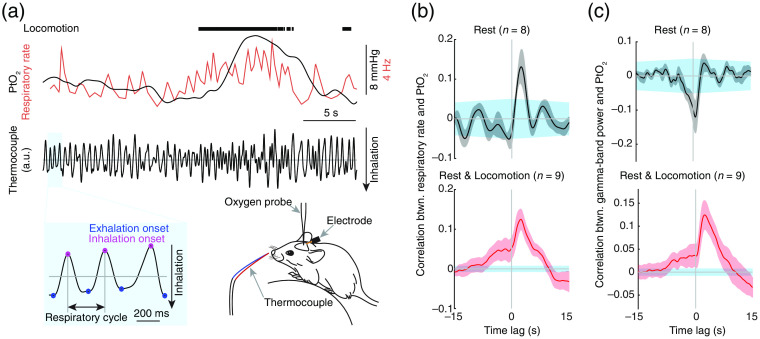
Respiration drives changes in cerebral tissue oxygenation. (a) Measuring respiration using a thermocouple. Measurements of breathing can be taken using thermocouples placed near the mouse’s nose (∼1  mm), with care taken to not contact the whiskers. Downward and upward deflections in respiration recordings correspond to inspiratory and expiratory phases of the respiratory cycle, respectively. The time of each expiratory peak in the entire record can be identified as the zero-crossing point of the first derivative of the thermocouple signal. During voluntary locomotion, respiratory rate increases. (b) Cross-correlation between brain tissue oxygenation (${\mathrm{PtO}}_{2}$) and respiratory rate during rest (top) and periods including rest and locomotion (bottom). (c) As (b) but for correlation between ${\mathrm{PtO}}_{2}$ and gamma-band LFP power. Data are shown as mean SEM in (b) and (c). Blue shaded region in (b) and (c) ~95 confidence interval of cross-correlation. Adapted from Ref. 8.

In studies involving humans or anesthetized animals, monitoring respiration usually uses an elastic belt or bioimpedance, with a calibration using spirometer. Monitoring respiration in awake animals is not an easy task, especially with additional simultaneous measurements, which makes plethysmography^154^ incompatible. As respiration is the result of volume changes in the lungs, resulting from respiratory muscle activity, which is the consequence of neuronal activity conveyed through nerves from the brain, we can monitor breathing using the neuronal activity of breathing circuit,^25^ the breathing muscle, and the air movement [see Ref. 154 for review]. Measuring the electromyography (EMG) of the diaphragm,^155^ the main inspiratory muscle, provides a good measurement of respiration, but is invasive and technically challenging. Measurement of respiration can be performed by measuring the cooling and warming of air arising from breathing using thermocouples.^6^^,^^8^^,^^156^ Although junction potentials and positioning differences mean the temperatures recorded are not quantitative indicators of airflow, the timing of inhalations and exhalations can be readily determined from the cooling-warming patterns [Fig. 9(a)]. In addition to the methods mentioned above, which are either invasive or require close contact with the animal, video monitoring is entirely noninvasive and contactless.^157^^,^^158^ However, the cost for this is significantly higher than previously mentioned methods. With the advance of high-speed cameras and the computer vision algorithms, video-based breath tracking should be cheap and easier in the future.

We use a small, externally positioned, temporally responsive thermocouple (40-gauge K-type thermocouple, TC-TT-K-40-36, Omega Engineering) to measure respiration rate^6^^,^^8^ (Fig. 9). The thermocouple is positioned ∼2  mm in front of and slightly inferior to the nostrils to maximize the temperature change in the thermocouple during expiration. The voltage changes generated by the temperature are amplified 2000×, filtered below 30 Hz (Model 440, Brownlee Precision), and sampled at 1000 Hz. Downward and upward deflections in respiration recordings correspond to inspiratory and expiratory phases of the respiratory cycle, respectively [Fig. 9(a)]. The time of each expiratory peak in the signal can be identified as the zero-crossing point of the first derivative of the thermocouple signal. One should take caution to make sure the thermocouple does not contact with the mouse whiskers or nostrils. It is noteworthy that the ambient temperature can significantly affect the amplitude of the signal from the thermocouple. The warmer the ambient air temperature, the smaller the amplitude of the voltage excursions caused by respiration. Mice move their nostrils during active sensing, and the amplitude of the voltage change during exhalation will depend on the direction the nostrils are pointed.^26^ Because of these effects, the absolute amplitude of the voltage change cannot be used to infer respiration depth.^159^

### 6.3 Monitoring Intracranial Pressure Changes during Behavior

The supply of blood to the brain depends on the cerebral perfusion pressure, i.e., the difference between the blood pressure and the ICP. Natural behaviors, such as locomotion,^61^ coughing,^160^ and sleep,^161^ all are accompanied by substantial increase in ICP. During voluntary locomotion, ICP increases in head-fixed mice^61^^,^^62^ and freely moving rats,^162^ and the increase of ICP precedes locomotion onset.^61^ Not only do these large movement cause significant increase of ICP, small movements, such as twitching and changes in body position,^111^ can increase ICP dramatically. It is important to understand the ICP change and its relation with different behaviors, because ICP elevation alters CSF clearance pathways,^163^^,^^164^ affects venous outflow dynamics^165^ and determines sympathetic activity.^166^^,^^167^ Increases in ICP are also associated with pathology. How ICP influences regulation of brain hemodynamics regulation and NVC is still debated. In anesthetized rats and baboons, moderate ICP elevation increases baseline CBF,^168^ while larger ICP elevations decrease baseline CBF.^169^ In response to somatosensory stimulation, post-stimulus deoxy-hemoglobin responses are attenuated by elevated ICP.^168^ Capillary RBC velocity in anesthetized rats does not change during ICP elevation.^170^ These differences are partially attributed to the method used to elevate ICP,^171^^,^^172^ which will change perfusion pressure and outflow resistance differently.

**Fig. 10:**
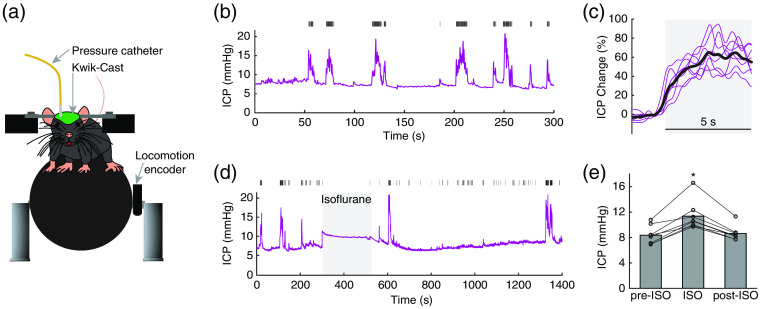
Monitoring ICP during locomotion and exposure to the vasodilator isoflurane. (a) Experimental setup. One week before the ICP measurement, a titanium head-bar is attached to the skull with cyanoacrylate glue and dental cement and the skull is covered with a thin layer of cyanoacrylate glue. After two days of recovery, the animal is habituated to head fixation on a spherical treadmill for one day (for three 30-min sessions, separated by 1 h in home cage with unrestricted access to food and water). On the day of the ICP experiment (one day after the habituation), the mouse is anesthetized with isoflurane and a small craniotomy (∼1-mm diameter) is made over the somatosensory cortex. A pressure measuring catheter (SPR-1000, Millar) is inserted into the cortex (1.0 mm caudal, 1-mm lateral from bregma), and a tight seal is made using Kwik-Cast (world precision instruments). This surgical procedure takes ∼10  min. Following the surgical procedure, the animal is allowed to wake from anesthesia and to freely locomote on the spherical treadmill for 2 h, during which both ICP and locomotion are recorded simultaneously at 1 kHz (NI USB-6003). To minimize any residual effect of anesthesia on ICP, we only analyze data collected more than 1 h after the cessation of anesthesia. (b) Example trace showing ICP dynamics during voluntary locomotion. Magenta trace shows the ICP changes, and black tick marks show locomotion events. (c) Averaged ICP changes in response to 5 s locomotion (gray shaded area). The thin lines show data from individual animals, and the thick line shows the group average. It is noteworthy that ICP rises before locomotion onset. (d) Example trace showing ICP dynamics before (pre-ISO), during (ISO) and after (post-ISO) isoflurane exposure. Isoflurane increases ICP immediately, and ICP recovers to baseline ∼2  min after isoflurane removal. Black tick marks show locomotion events. The ICP increases during isoflurane are smaller than those accompanying locomotion. (e) Isoflurane exposure significantly increases ICP. Adapted from Ref. 61.

ICP can be measured at different sites within the brain, with intraventricular and intraparenchymal measurements being most common.^173^^,^^174^ Here, we introduce our intraparenchymal ICP measurement protocol in awake, head-fixed mice^61^^,^^62^ (Fig. 10). Before the ICP measurement, we implant a head-bar, and habituate the mouse running on a spherical treadmill for at least three days. Before each experiment, the pressure sensor (model SPR1000; diameter, 0.33 mm; Millar, Houston, Texas) is stabilized by soaking the sensor in room-temperature sterile water overnight, and is calibrated using the built-in function of the pressure control unit. On the day of ICP measurements, the mouse was anesthetized and a hole 0.5 mm in diameter is drilled in the skull above the left parietal lobe. The pressure sensor is inserted through the hole perpendicularly into the cortex to a depth of 1 mm. The probe is sealed in place with Kwik-Cast (World Precision Instruments). Placing the probe perpendicularly into the brain, rather than between the skull and pia, reduces damage to the pial vascular network that supplies the cortex.^175^ Control experiments, in which the ICP probe was vibrated in water, showed that rapid motion does not cause detectable pressure changes, and an ICP probe implanted in a dead mouse did not register any ICP changes when the ball was rotated.^61^

## 7 Cortical State Monitoring: Detecting Sleep

Head-fixed mice will fall asleep, and because sleep in mice can be highly fragmented, sleep periods lasting tens of seconds to minutes can be interspersed with wake periods of comparable duration.^176^^,^^177^ Examples of sleep-related changes in CBV are shown in Fig. 4. In a single-vessel scale (Fig. S2 in the Supplemental Material), during NREM sleep, arteriole diameter follows a low-frequency dilation/constriction with peak dilations that can exceed those seen during moderate whisking (Fig. 3). During REM sleep, the arterioles slowly dilate over tens of seconds, and the dilation is substantially larger than anything that occurs in the awake animal^37^ (Fig. S2 in the Supplemental Material). Therefore, if sleep is present, it will greatly impact or even dominate any neurovascular signals.

The large impact of sleep on neurovascular signals means that periods of sleep need to be detected to avoid contamination. This can be done by “sleep scoring,” in which physiological and behavioral signals are used to determine if the mouse is awake, or in NREM or REM sleep. Sleep scoring^153^^,^^178^ has been extensively used on both humans and animals, and the criteria will differ from species to species. While a detailed presentation of sleep scoring methodology is beyond the scope of this review, using the behavioral and physiological observation techniques here can help to determine if sleep is occurring during functional brain imaging. In general, mice do not make any bodily motions during sleep, though they sometimes do on arousal. There is essentially no whisker movement during NREM sleep (Fig. 4 and Fig. S2 in the Supplemental Material), so it is highly probable that the mouse has fallen asleep after >20  s of whisker immobility. The whiskers do move during REM sleep (Fig. 4 and Fig. S2 in the Supplemental Material), but REM sleep is almost always preceded by periods of NREM sleep. During NREM sleep there is a broadband increase in the cortical^37^^,^^179^^,^^180^ and hippocampal^37^^,^^181^^,^^182^ LFP power, which is particularly large in the lower (∼1 to 4 Hz) frequency bands (Figs. 4 and 7). This is in contrast to the cortical LFP changes during locomotion and other movements, where power in the lower frequencies of the LFP (1 to 4 Hz) goes down (Fig. 6). The heart rate is low during NREM sleep (∼6  Hz), somewhat higher during REM (∼7 to 8 Hz), and is highest on average (but more variable), in the awake state (Fig. 4). Pupil diameter is qualitatively different during different sleep states. During REM sleep, the pupil is very constricted, while during NREM sleep, the pupil’s diameter fluctuates, though the diameter is smaller than in the awake states^38^ (Fig. 4). Finally, during sleep there are large changes in arterial diameter and blood volume (Fig. 4 and Fig. S2 in the Supplemental Material). During NREM sleep, arteriole diameter undergoes low frequency (0.01 to 0.1 Hz) oscillations with peak dilations of up to 30% above baseline. During REM sleep, the arterioles slowly dilate up to 50%.^37^ In general, arterial dilations of more than 10% in the absence of sensory stimulation that are not accompanied by body/whisker movement are hallmarks of the mouse falling asleep. Anecdotally, we very rarely see sleep with mice on spherical treadmills (Fig. S1 in the Supplemental Material), but it is much more common when mice are head-fixed and restrained in a tube (Fig. 4 and Fig. S2 in the Supplemental Material). Lighting matters as well. Because mice are nocturnal, they are more likely to sleep with visible light present, e.g., during IOS imaging.

## 8 Discussion

Behavior is an important determinant of neural activity and hemodynamic changes in un-anesthetized animals. Under many conditions, spontaneous movements and arousal changes can account for most of the noise or spontaneous fluctuations in neural and vascular responses. Without monitoring behavior and arousal state, any evoked signals may be swamped, and differences in arousal level and spontaneous motion (e.g., due to age, drug treatment, or other manipulation) could confound comparisons of neurovascular responses across groups. However, tools to address behavioral confounds are inexpensive and readily available. We believe that the adoption of routine monitoring of body position and physiological manifestations of arousal will increase the rigor of research, help comparisons across laboratories, and to give a better understanding of the origins of neural and vascular signals.

## 9 Code, Data, and Materials Availability

Source code and example data used in this tutorial is available in the online repositories without access restrictions under a general public license at DrewLab Github (https://github.com/DrewLab).
