# Supplementary material for: Behavioral and physiological monitoring for awake neurovascular coupling experiments: a how-to guide
Source: Neurophotonics. 2022 Jan 27;9(2):021905. doi: 10.1117/1.NPh.9.2.021905 (PMC8802326; doi:10.1117/1.NPh.9.2.021905)
Supplement: Supplementary file 1 [file NPh_009_021905_SD001.pdf]

a

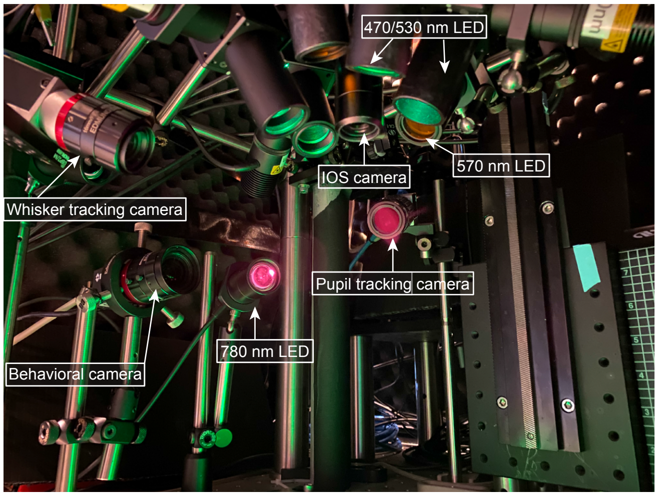
470/530 nm LED
IOS camera
570 nm LED
Whisker tracking camera
Pupil tracking camera
Behavioral camera
780 nm LED

b

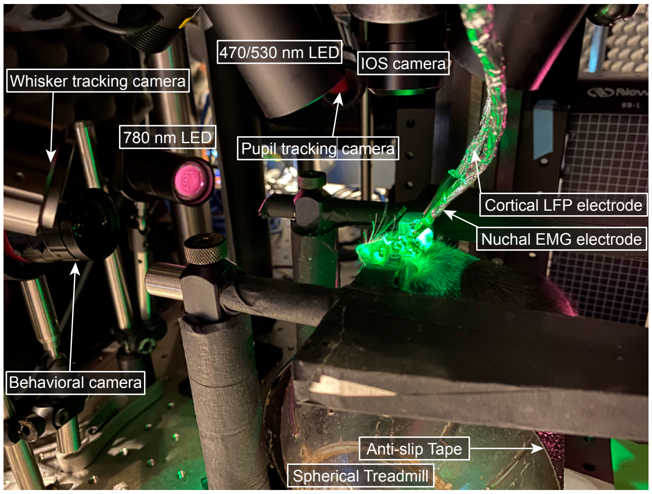
470/530 nm LED
IOS camera
Whisker tracking camera
780 nm LED
Pupil tracking camera
Cortical LFP electrode
Nuchal EMG electrode
Behavioral camera
Anti-slip Tape
Spherical Treadmill

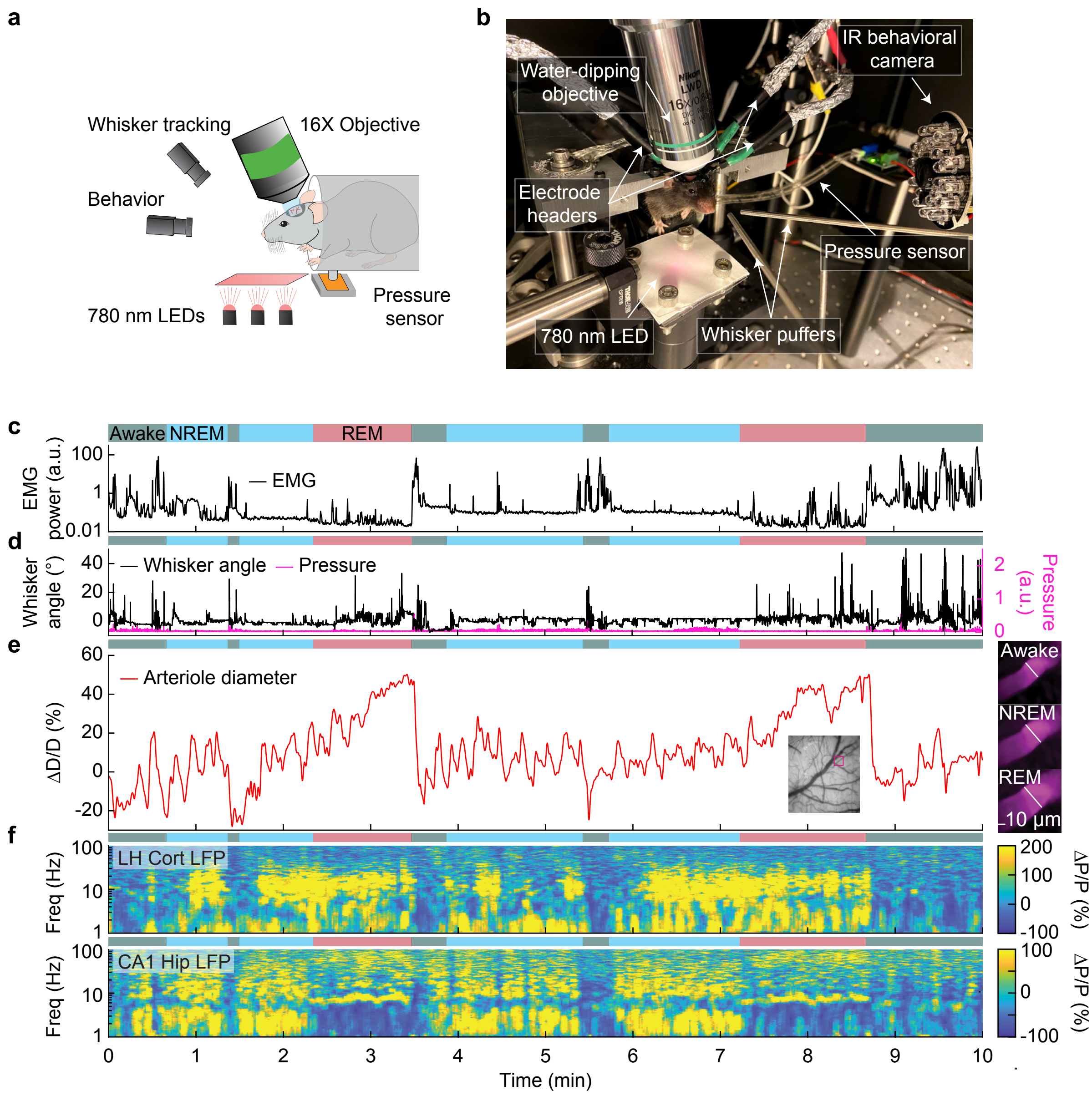
a
Whisker tracking
16X Objective
Behavior
780 nm LEDs
Pressure sensor
b
Water-dipping objective
IR behavioral camera
Electrode headers
Pressure sensor
780 nm LED
Whisker puffers
c
Awake
NREM
REM
EMG power (a.u.)
EMG
d
Whisker angle (°)
Whisker angle
Pressure
Pressure (a.u.)
e
ΔD/D (%)
Arteriole diameter
Awake
NREM
REM
10 μm
f
Freq (Hz)
LH Cort LFP
ΔP/P (%)
CA1 Hip LFP
Time (min)

Function	Parts	Manufacture
**Thin-skull window illumination**		
Mounted LEDs	M530L3	Thorlabs
	M565L3	Thorlabs
	M660L3	Thorlabs
	M470L3	Thorlabs
Collimating lens for LEDs	KPX076	Newport
Filters	FB530-10	Thorlabs
	FB570-10	Thorlabs
	FB470-10	Thorlabs
LED drivers	LEDD1B	Thorlabs
Dichroic Beamsplitter	FF506-Di03-25x36	Semrock
**Brain hemodynamics imaging**		
Camera	Dalsa 1M60	Teledyne
Lens	VZM 300i	Edmund Optics
**Pupil tracking**		
Illumination	M780L3	Thorlabs
Camera	acA640-120gm	Basler
Lens	#67-714	Edmund Optics
**Behavior monitoring**		
Illumination	M780L3	Thorlabs
Facial camera	FLIR: BFS-U3-51S5M-C	Teledyne
Optical encoder	E7PD-720-118	US Digital
Force sensor	Flexiforce A201	Tekscan
Whisker illumination	#66-833	Edmund Optics
Whisker camera	acA640-120gm	Basler
**Mouse head-fixation**		
Head bar	custom	https://github.com/DrewLab/Mouse-Head-Fixation
Head bar holder	custom	
**Respiration monitoring**		
Thermocouples	TC-TT-K-40-36	Omega Engineering
**Electrophysiology**		
Amplifier	DAM80	World Precision Instruments
	Model 440	Brownlee Precision
**Data I/O**		
Multifunction I/O device	PCI-6259	National Instruments
	USB-6343	National Instruments
Frame grabber device	PCI-1428	National Instruments
Terminal block	BNC-2090A	National Instruments
